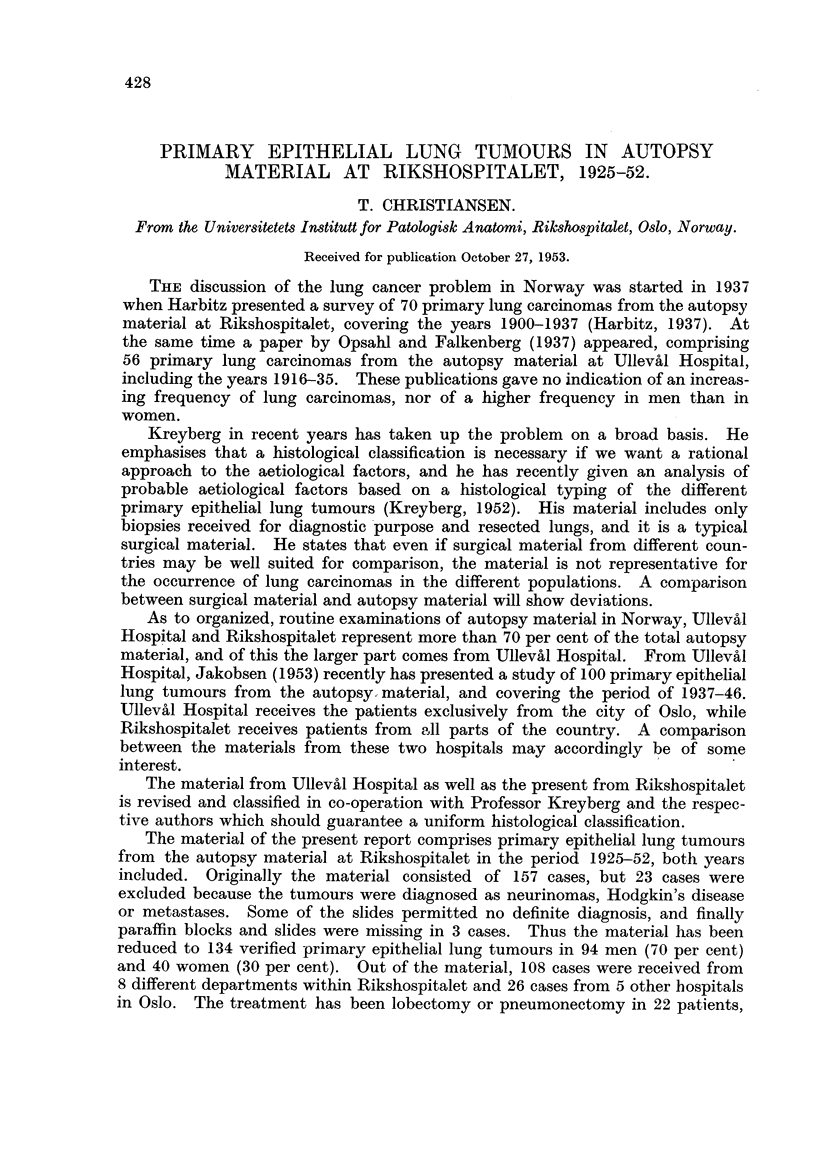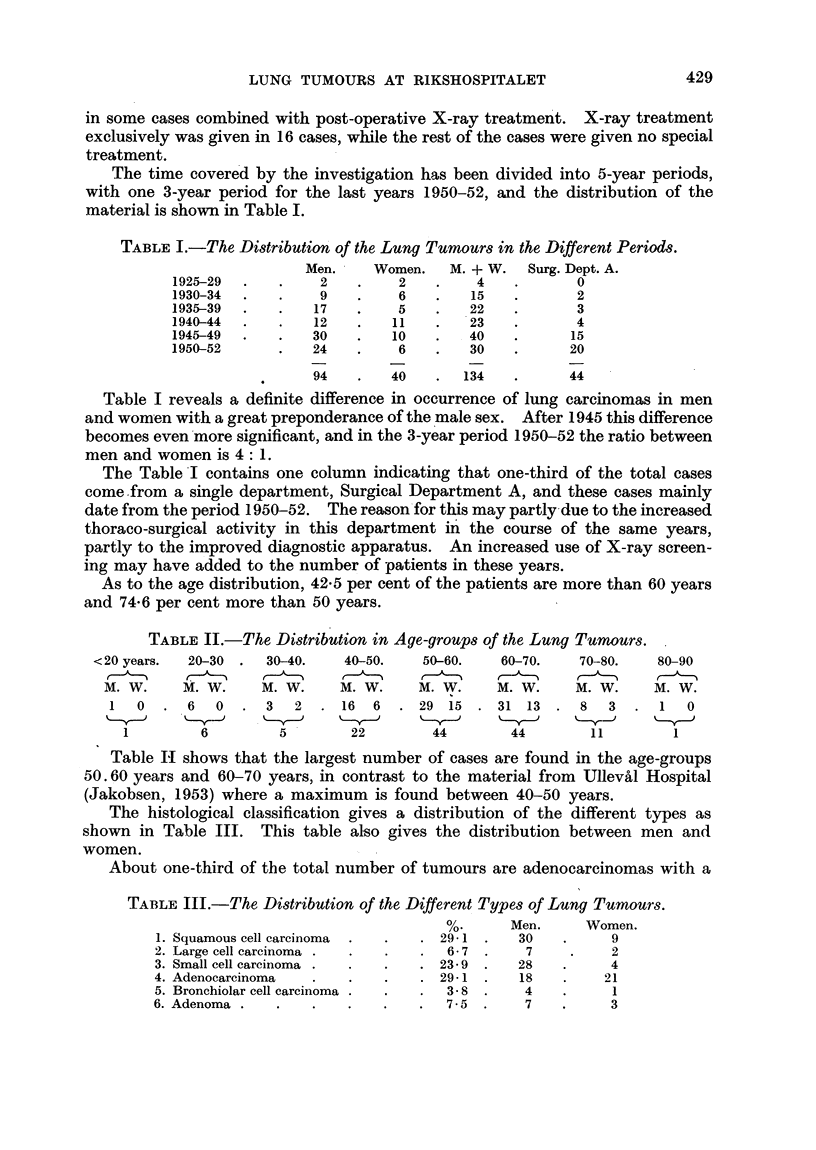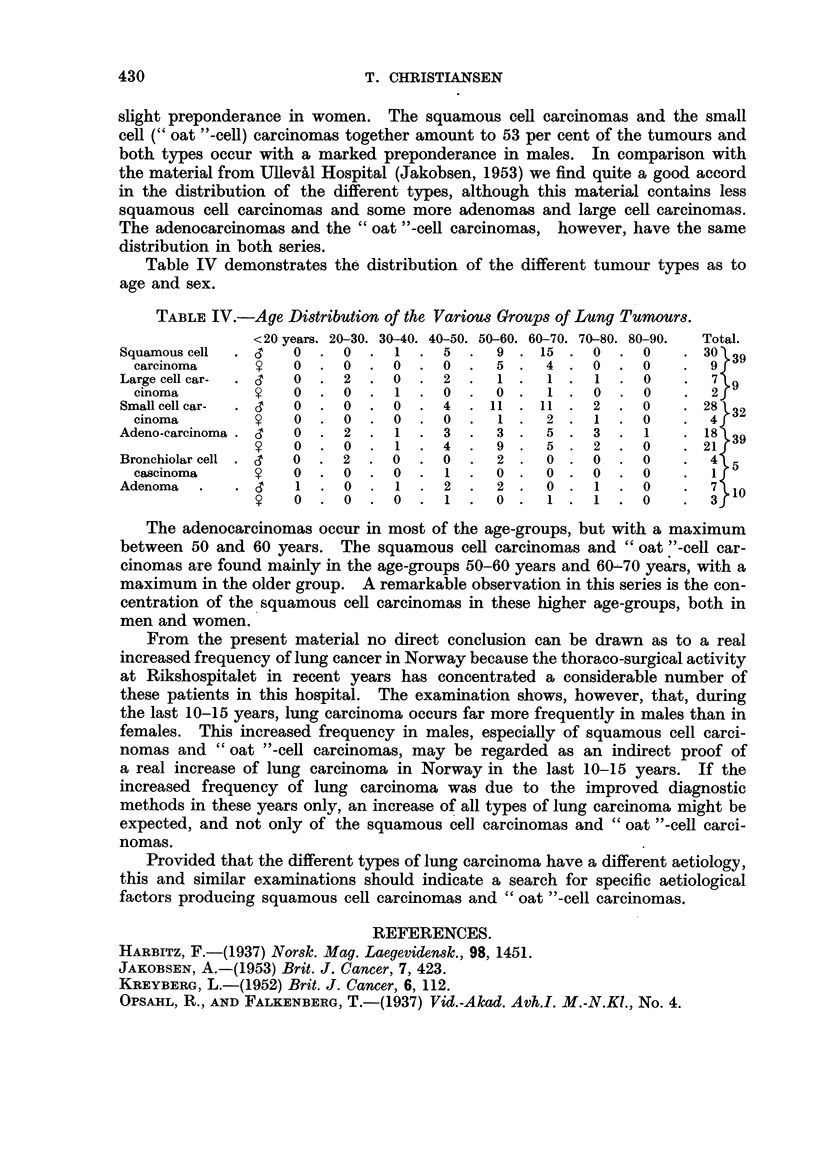# Primary Epithelial Lung Tumours in Autopsy Material at Rikshospitalet, 1925-52

**DOI:** 10.1038/bjc.1953.42

**Published:** 1953-12

**Authors:** T. Christiansen


### PRIMARY EPITHELIAL LUNG TUMOURS IN AUTOPSY MATERIAL AT RIKSHOSPITALET, 1925–52.

T. CHRISTIANSEN.

*From the Universitetets Institutt for Patologisk Anatomi, Rikshospitalet, Oslo, Norway.*

Received for publication October 27, 1953.

THE discussion of the lung cancer problem in Norway was started in 1937 when Harbitz presented a survey of 70 primary lung carcinomas from the autopsy material at Rikshospitalet, covering the years 1900–1937 (Harbitz, 1937). At the same time a paper by Opsahl and Falkenberg (1937) appeared, comprising 56 primary lung carcinomas from the autopsy material at Ullevål Hospital, including the years 1916–35. These publications gave no indication of an increasing frequency of lung carcinomas, nor of a higher frequency in men than in women.

Kreyberg in recent years has taken up the problem on a broad basis. He emphasises that a histological classification is necessary if we want a rational approach to the aetiological factors, and he has recently given an analysis of probable aetiological factors based on a histological typing of the different primary epithelial lung tumours (Kreyberg, 1952). His material includes only biopsies received for diagnostic purpose and resected lungs, and it is a typical surgical material. He states that even if surgical material from different countries may be well suited for comparison, the material is not representative for the occurrence of lung carcinomas in the different populations. A comparison between surgical material and autopsy material will show deviations.

As to organized, routine examinations of autopsy material in Norway, Ullevål Hospital and Rikshospitalet represent more than 70 per cent of the total autopsy material, and of this the larger part comes from Ullevål Hospital. From Ullevål Hospital, Jakobsen (1953) recently has presented a study of 100 primary epithelial lung tumours from the autopsy material, and covering the period of 1937–46. Ullevål Hospital receives the patients exclusively from the city of Oslo, while Rikshospitalet receives patients from all parts of the country. A comparison between the materials from these two hospitals may accordingly be of some interest.

The material from Ullevål Hospital as well as the present from Rikshospitalet is revised and classified in co-operation with Professor Kreyberg and the respective authors which should guarantee a uniform histological classification.

The material of the present report comprises primary epithelial lung tumours from the autopsy material at Rikshospitalet in the period 1925–52, both years included. Originally the material consisted of 157 cases, but 23 cases were excluded because the tumours were diagnosed as neurinomas, Hodgkin's disease or metastases. Some of the slides permitted no definite diagnosis, and finally paraffin blocks and slides were missing in 3 cases. Thus the material has been reduced to 134 verified primary epithelial lung tumours in 94 men (70 per cent) and 40 women (30 per cent). Out of the material, 108 cases were received from 8 different departments within Rikshospitalet and 26 cases from 5 other hospitals in Oslo. The treatment has been lobectomy or pneumonectomy in 22 patients, in some cases combined with post-operative X-ray treatment. X-ray treatment exclusively was given in 16 cases, while the rest of the cases were given no special treatment.

The time covered by the investigation has been divided into 5-year periods, with one 3-year period for the last years 1950–52, and the distribution of the material is shown in Table I.

TABLE I.—*The Distribution of the Lung Tumours in the Different Periods.*

| | Men. | Women. | M. + W. | Surg. Dept. A. |
|---|---|---|---|---|
| 1925–29 | 2 | 2 | 4 | 0 |
| 1930–34 | 9 | 6 | 15 | 2 |
| 1935–39 | 17 | 5 | 22 | 3 |
| 1940–44 | 12 | 11 | 23 | 4 |
| 1945–49 | 30 | 10 | 40 | 15 |
| 1950–52 | 24 | 6 | 30 | 20 |
| | 94 | 40 | 134 | 44 |

Table I reveals a definite difference in occurrence of lung carcinomas in men and women with a great preponderance of the male sex. After 1945 this difference becomes even more significant, and in the 3-year period 1950–52 the ratio between men and women is 4 : 1.

The Table I contains one column indicating that one-third of the total cases come from a single department, Surgical Department A, and these cases mainly date from the period 1950–52. The reason for this may partly due to the increased thoraco-surgical activity in this department in the course of the same years, partly to the improved diagnostic apparatus. An increased use of X-ray screening may have added to the number of patients in these years.

As to the age distribution, 42·5 per cent of the patients are more than 60 years and 74·6 per cent more than 50 years.

TABLE II.—*The Distribution in Age-groups of the Lung Tumours.*

| <20 years. | 20–30 | 30–40. | 40–50. | 50–60. | 60–70. | 70–80. | 80–90 |
|---|---|---|---|---|---|---|---|
| M. W. | M. W. | M. W. | M. W. | M. W. | M. W. | M. W. | M. W. |
| 1 0 | 6 0 | 3 2 | 16 6 | 29 15 | 31 13 | 8 3 | 1 0 |
| 1 | 6 | 5 | 22 | 44 | 44 | 11 | 1 |

Table II shows that the largest number of cases are found in the age-groups 50.60 years and 60–70 years, in contrast to the material from Ullevål Hospital (Jakobsen, 1953) where a maximum is found between 40–50 years.

The histological classification gives a distribution of the different types as shown in Table III. This table also gives the distribution between men and women.

About one-third of the total number of tumours are adenocarcinomas with a

TABLE III.—*The Distribution of the Different Types of Lung Tumours.*

| | %. | Men. | Women. |
|---|---|---|---|
| 1. Squamous cell carcinoma | 29·1 | 30 | 9 |
| 2. Large cell carcinoma | 6·7 | 7 | 2 |
| 3. Small cell carcinoma | 23·9 | 28 | 4 |
| 4. Adenocarcinoma | 29·1 | 18 | 21 |
| 5. Bronchiolar cell carcinoma | 3·8 | 4 | 1 |
| 6. Adenoma | 7·5 | 7 | 3 |

slight preponderance in women. The squamous cell carcinomas and the small cell (" oat "-cell) carcinomas together amount to 53 per cent of the tumours and both types occur with a marked preponderance in males. In comparison with the material from Ullevål Hospital (Jakobsen, 1953) we find quite a good accord in the distribution of the different types, although this material contains less squamous cell carcinomas and some more adenomas and large cell carcinomas. The adenocarcinomas and the " oat "-cell carcinomas, however, have the same distribution in both series.

Table IV demonstrates the distribution of the different tumour types as to age and sex.

TABLE IV.—*Age Distribution of the Various Groups of Lung Tumours.*

| | | <20 years. | 20–30. | 30–40. | 40–50. | 50–60. | 60–70. | 70–80. | 80–90. | Total. | |
|---|---|---|---|---|---|---|---|---|---|---|---|
| Squamous cell carcinoma | ♂ | 0 | 0 | 1 | 5 | 9 | 15 | 0 | 0 | 30 | 39 |
| | ♀ | 0 | 0 | 0 | 0 | 5 | 4 | 0 | 0 | 9 | |
| Large cell carcinoma | ♂ | 0 | 2 | 0 | 2 | 1 | 1 | 1 | 0 | 7 | 9 |
| | ♀ | 0 | 0 | 1 | 0 | 0 | 1 | 0 | 0 | 2 | |
| Small cell carcinoma | ♂ | 0 | 0 | 0 | 4 | 11 | 11 | 2 | 0 | 28 | 32 |
| | ♀ | 0 | 0 | 0 | 0 | 1 | 2 | 1 | 0 | 4 | |
| Adeno-carcinoma | ♂ | 0 | 2 | 1 | 3 | 3 | 5 | 3 | 1 | 18 | 39 |
| | ♀ | 0 | 0 | 1 | 4 | 9 | 5 | 2 | 0 | 21 | |
| Bronchiolar cell cascinoma | ♂ | 0 | 2 | 0 | 0 | 2 | 0 | 0 | 0 | 4 | 5 |
| | ♀ | 0 | 0 | 0 | 1 | 0 | 0 | 0 | 0 | 1 | |
| Adenoma | ♂ | 1 | 0 | 1 | 2 | 2 | 0 | 1 | 0 | 7 | 10 |
| | ♀ | 0 | 0 | 0 | 1 | 0 | 1 | 1 | 0 | 3 | |

The adenocarcinomas occur in most of the age-groups, but with a maximum between 50 and 60 years. The squamous cell carcinomas and " oat "-cell carcinomas are found mainly in the age-groups 50–60 years and 60–70 years, with a maximum in the older group. A remarkable observation in this series is the concentration of the squamous cell carcinomas in these higher age-groups, both in men and women.

From the present material no direct conclusion can be drawn as to a real increased frequency of lung cancer in Norway because the thoraco-surgical activity at Rikshospitalet in recent years has concentrated a considerable number of these patients in this hospital. The examination shows, however, that, during the last 10–15 years, lung carcinoma occurs far more frequently in males than in females. This increased frequency in males, especially of squamous cell carcinomas and " oat "-cell carcinomas, may be regarded as an indirect proof of a real increase of lung carcinoma in Norway in the last 10–15 years. If the increased frequency of lung carcinoma was due to the improved diagnostic methods in these years only, an increase of all types of lung carcinoma might be expected, and not only of the squamous cell carcinomas and " oat "-cell carcinomas.

Provided that the different types of lung carcinoma have a different aetiology, this and similar examinations should indicate a search for specific aetiological factors producing squamous cell carcinomas and " oat "-cell carcinomas.

### REFERENCES.

HARBITZ, F.—(1937) *Norsk. Mag. Laegevidensk.*, **98**, 1451.
JAKOBSEN, A.—(1953) *Brit. J. Cancer*, **7**, 423.
KREYBERG, L.—(1952) *Brit. J. Cancer*, **6**, 112.
OPSAHL, R., AND FALKENBERG, T.—(1937) *Vid.-Akad. Avh.I. M.-N.Kl.*, No. 4.